# A Group-Administered social Skills Training for 8- to 12- Year-Old, high-Functioning Children With Autism Spectrum Disorders: An Evaluation of its Effectiveness in a Naturalistic Outpatient Treatment Setting

**DOI:** 10.1007/s10803-016-2887-1

**Published:** 2016-08-13

**Authors:** Anne Deckers, Peter Muris, Jeffrey Roelofs, Arnoud Arntz

**Affiliations:** 1 Virenze RIAGG Maastricht (Child and Youth Care) and Department of Clinical Psychological Science, Maastricht University, Postbox 616, 6200 MD Maastricht, The Netherlands; 2 Department of Clinical Psychology, University of Amsterdam, Amsterdam, The Netherlands

**Keywords:** Social skills, Autism spectrum disorders, Children

## Abstract

A social skills training (SST) for high-functioning children with autism spectrum disorders (ASD) was evaluated in an outpatient setting using a combined between- and within-subject design in which SST and a waiting list condition were compared. According to parents and teachers, the SST produced greater improvement of social skills than the waiting list, and these effects were maintained at 3 months follow-up. No between-group effects were found for loneliness, although in general scores on this outcome measure decreased from pre- to follow-up. The effects of SST were unaffected by social anxiety, ADHD symptoms, Theory of Mind, or desire for social interaction. Altogether, SST seems an effective intervention for high-functioning children with ASD that can be applied in daily clinical practice.

## Introduction

Engaging in social interaction is an inevitable and significant element of daily life. An extensive line of research has shown that positive interpersonal relationships are important for both the physical and emotional welfare of human beings (Baumeister and Leary 1995). However, for children with autism spectrum disorders (ASD) such relationships can by no means be taken for granted because of their significant deficits in communication and social interaction (American Psychiatric Association 2013). Impaired social skills are a core feature of ASD (Rao et al. 2008) and an increasing number of studies has demonstrated that children with ASD encounter elevated levels of social difficulties in their daily lives. For example, Calder et al. (2013) found that children with ASD have fewer reciprocal friendships and lower friendship quality as compared to their peers. In addition, children with ASD are more isolated and have a less central position in social networks (Kasari et al. 2011; Rotheram-Fuller et al. 2010). Furthermore, children with ASD are more often a victim of bullying (Rowley et al. 2012). The finding that children with ASD also report elevated levels of loneliness (e.g., Bauminger and Kasari 2000) suggests that they are not satisfied with their social functioning. Finally, the importance of social skills is not restricted to social functioning but also affects the educational progress of children and as such has a long-term impact on occupational functioning and well-being in later life (Hartup 1989; Howlin et al. 2004).

Social skills training (SST) is one of the interventions that can be applied in order to facilitate socialization in children with ASD (Rogers 2000). This type of intervention is preferably provided in a group format because of the convenience of naturally occurring interactions and practicing opportunities with peers (Lopata et al. 2008). Despite the widespread application of group SST for children with ASD–especially for those who are high-functioning–the empirical evidence for this type of intervention is still limited (Reichow and Volkmar 2010). In their review, Rao et al. (2008) concluded that the majority of the 10 studies so far conducted in high-functioning children with ASD have documented positive outcomes for this type of intervention. However, the authors also noted that most of this research suffers from methodological limitations such as lack of standardized treatment manuals, small sample sizes, absence of control groups, and no inclusion of follow-up assessments. In a similar vein, Reichow et al. (2012) systematically reviewed the evidence for the effectiveness of SST in youth with ASD and identified only five RCTs. They pointed at the limited amount of research, but also noted findings that were quite encouraging for clinical practice as this type of intervention appears to promote social competence and friendships, while decreasing feelings of loneliness. Another important shortcoming of previous research on the effects of SST concerns the generalization of social skills outside the treatment setting. Obviously, the ultimate goal of this type of intervention is that children with ASD are able to deploy the newly acquired social skills in social situations such as at home and in school. Most studies to date employed SST interventions that did not include strategies to enhance this type of generalization, or did not include a measurement for evaluating whether and to what extent the trained social skills actually generalize outside the treatment setting. Rao et al. (2008) strongly recommended that future research in this area should make the effort to promote generalization of SST and to measure its effects in everyday social situations outside the therapeutic setting (see also Krasny et al. 2003; Williams White et al. 2007).

Children with ASD constitute a very heterogeneous group with variable clinical and psychological features. It may well be that a number of these features have an impact on the efficacy of a group SST intervention. A first characteristic concerns the presence of comorbid psychiatric symptoms (e.g., Mattila et al. 2010; Simonoff et al. 2008; Steensel et al. 2013a), of which social anxiety and ADHD seem particularly relevant. For instance, it has been demonstrated that high levels of social anxiety are linked to lower levels of social functioning (Chang et al. 2012), and it is also suggested that this relation is bidirectional (Bellini 2006). From this one might expect that children with ASD and high social anxiety will profit less from SST. The latter could also be true for children with ASD and comorbid ADHD as inattention may interfere with the learning of social skills, hyperactivity may disrupt their functioning in the group sessions, and impulsivity may hinder the application of the acquired abilities in daily situations. Interestingly, Antshel et al. (2011) examined the influence of these common psychiatric comorbidities on group SST outcomes for children with ASD. As hypothesized, it was found that group SST was less effective in children with comorbid ASD and ADHD (all subtypes combined): the social skills of these children did not improve over the course of the treatment. It was surprising to see, however, that children with comorbid ASD and anxiety disorders profited equally from this type of intervention when compared to children with ASD alone. Apparently, “the structured group setting and the focus on social problem solving are well suited to the needs of children with ASD [and anxiety disorders]” (Antshel et al. 2011; p. 444), so that their comorbidity was no obstacle for a positive response to group SST. Altogether, research suggests that group SST is a valuable intervention for children with ASD even when a comorbid anxiety disorder is present, however group SST seems less effective in ASD children with comorbid ADHD. Before definitively accepting this conclusion, more research is required.

A second feature that might influence the efficacy of group SST for children with ASD concerns the developmental level of Theory of Mind (ToM). ToM has been defined as the ability to ascribe thoughts, feelings, ideas, and intentions to others and to employ this ability to anticipate the behavior of others (Premack and Woodruff 1978). ToM is generally seen as important for understanding the social environment and for engaging in socially competent behavior (Wellman 1990). It has been proposed (e.g., Baron-Cohen 2000; Baron-Cohen et al. 1985) that the social impairments seen in children with ASD are due to marked deficits in their ToM. From this, it can be hypothesized that the level of ToM may be a significant predictor of the outcome of SST for children with ASD. More specifically, given that there are clear individual differences in ToM ability across children with ASD, it may well be that children with ASD and severe ToM deficits will profit less from SST than children with ASD who have relatively high levels.

Finally, interest and motivation are important requirements for learning (e.g., Krapp 1999), and this also seems to apply to the acquisition of social skills (e.g., Van Doesem et al. 2013). Chevallier et al.(2012) have put forward the social motivation theory of autism, which implies that the social problems of children with ASD can be traced back to the lack of intrinsic desire to interact with others, and there is indeed tentative empirical support for this notion (Deckers et al. 2014). So it may well be the case that the desire for social interaction of children with ASD is an important moderator of the treatment effects of a SST intervention. That is, if children with ASD have too little desire to engage in social interactions, they will probably be less motivated for participating in this type of training, which in turn may seriously interfere with the acquisition of social skills. In contrast, children with ASD who have a strong desire for social interaction may be more responsive to SST.

The purpose of the present naturalistic clinical study was to evaluate a group SST for 8- to 12 year-old, high-functioning children with ASD in an outpatient treatment setting. Effort was made to implement the essential ingredients and requirements of this type of intervention, which have been described in detail by (Krasny et al. 2003). So, the most important aim of this group SST was not to improve social skills in the clinical setting, but to promote transference of such abilities in order to enhance social functioning in daily life. Further, a standardized training manual was employed which facilitates implementation in other clinical settings as well as replication of the research. Treatment outcome was evaluated using a multi-informant approach that included parents and teachers who were asked to rate the social skills of the children based on observations at home and in school. This enabled us to measure the generalization of treatment effects in daily life. In order to evaluate the effect of the SST on the perception of their own social functioning, children completed a scale measuring loneliness as a secondary outcome measure. Finally, the study also included a waiting list control condition against which the effects of the group SST were compared.

The study set out to test a number of hypotheses: (1). On the between-group level, children in the group SST condition were expected to show a larger increase in parent- and teacher-rated social skills (i.e., the primary outcome measure) as compared to children in the waiting list control condition (WLC); (2). In addition, children in the SST condition would show a larger decrease in loneliness (i.e., the secondary outcome measure) as compared to children in the WLC; (3). On the within-subjects level, both parent- and teacher-rated social skills would improve, whereas child-reported loneliness would decline following the group SST; (4). The positive effects of SST, where found, were expected to be still visible at the follow-up assessment; and (5). Comorbid symptoms, in particular those related to ADHD, would have a negative influence on treatment outcome, whereas a more advanced level of ToM and a stronger desire for social interaction would have a positive impact on the effect of the group SST.

## Method

### Design

**Fig. 1:**
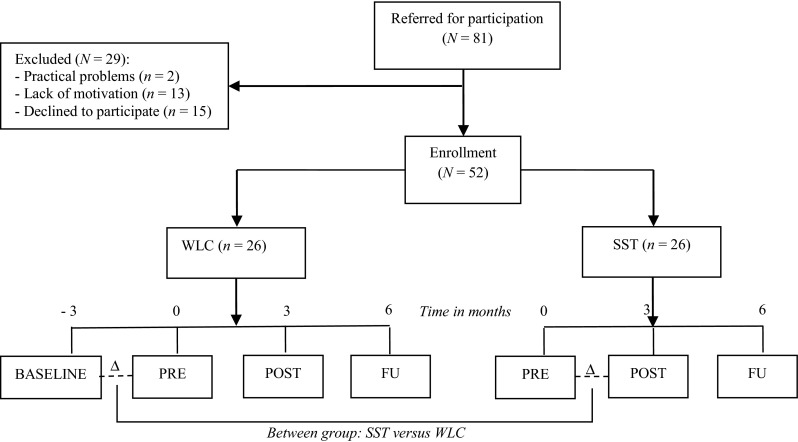
Enrollment and allocation of the participants and a visual representation of the study design (assessment points are displayed for both conditions). Note. *WLC* waiting list condition, *SST* social skills training

The group SST was evaluated in an outpatient treatment setting with clinically referred children. A combined between- and within-subjects design was applied. Half of the participants were first on a natural waiting list condition (WLC) before the group SST started, while the other half of the participants immediately started with the group SST. This implies that participants in the WLC were measured on four time points, whereas the other participants were assessed on three time points. The first assessment of the WLC took place 3 months prior to the start of the group SST (BASELINE). Both groups of participants were measured immediately prior to the group SST (PRE), directly after this intervention (POST), and at 3 months follow-up (FU; see Fig. 1). Multiple informants were involved in the assessments conducted for this study and included children, parents, and teachers.

### Procedure

Participants were recruited at a community mental health center (Virenze-RIAGG Maastricht, the Netherlands). The inclusion criteria were a formal diagnosis of Pervasive Developmental Disorder-Not Otherwise Specified (PDD-NOS), Asperger’s Disorder, or Autistic Disorder; an age between 8 and 12 years; and the absence of severe cognitive and language impairments. The presence of comorbid (psychiatric) diagnoses was not an exclusion criterion. All children who met these criteria according to the specialized, multidisciplinary team of the community mental health center and, who were indicated for participation in the group SST by this team were invited to participate. Some children were placed on a waiting list, as groups of four children were formed (children’s chronological and mental age were taken into account when composing the groups in order to maximize interpersonal match) and therapists were not always directly available to run the group. In addition, there were time constraints: we wanted to deliver the SST during 12 consecutive weeks, without a disruption by school holidays. Children who had to wait 3 months, were assessed again prior to the start of the intervention, and thus formed a natural WLC. Children who did not have to wait, were assessed for the first time and then entered the SST group shortly after. Thus, the allocation to either the WLC or SST condition can be considered as quasi-random. Because of ethical considerations, additional types of treatment for the child and/or the parents were allowed prior (thus, also during wait) or in parallel with the group SST.

For each child, the formal DSM-IV-TR diagnosis (American Psychiatric Association 2000) was established according to the Longitudinal-Expert-All Data (LEAD) principle (Spitzer 1983). A specialized multidisciplinary team consisting of licensed child psychologists and child psychiatrists made the classification on the basis of extensive assessments, using multiple informants during a longitudinal diagnostic process (Roelofs et al. 2015). More precisely, a clinical interview was carried out with parents and the child to identify the presence of psychopathology in general. In addition, a diagnostic interview specifically focusing on autism spectrum characteristics was completed with the parents to explore the developmental history of the child, and its current social functioning, communication, and behavior. Teachers were also interviewed regarding the child and his/her interactions with peers, communication, behavior, and didactic functioning in school. In addition, the child was observed by a psychologist or psychiatrist in a playroom setting. In case of educational delays and/or suspicion of limited cognitive abilities of the child, an IQ test was administered.

### Participants

Of 81 children eligible for group SST, 29 children did not participate due to practical problems, lack of motivation, or because they did not want to participate in research (see Fig. 1). The final sample hence consisted of 52 children (47 boys and 5 girls) with ASD, including (high-functioning) Autistic Disorder (*n* = 4), Asperger’s disorder (*n* = 13), and PDD-NOS (*n* = 35; see Table 1). The different types of ASD were equally distributed across the SST and WLC. The two groups did not differ in terms of parent-rated autistic behaviors, as measured with the Children’s Social Behavior Questionnaire (CSBQ; Luteijn et al. 2002; *t*(49) < 1). For the total sample, the mean score on the CSBQ was 42.6 (*SD* = 14.46), indicating symptoms levels in the clinical range (Van Steensel et al. 2015).

**Table 1:** Demographic and clinical characteristics of the total sample of ASD children and a comparison between the two conditions

	Total sample (*N* = 52)	WLC (*n* = 26)	SST (*n* = 26)	χ^2^	*P*
*Gender*				.221	.638
Male	47	23	34		
Female	5	3	2		
*Education*				1.038	.308
Regular	41	22	19		
Special	11	4	7		
*Diagnosis*				.105	.949
Autism	4	2	2		
Asperger	13	6	7		
PDD-NOS	35	18	17		
*Medication*				.325	.569
Yes	20	9	11		
No	32	17	15		
*Comorbidity*				2.836	.092
Present	30	18	12		
Absent	22	8	14		
*Comorbid* *ADHD*				1.997	.158
Present	21	13	8		
Absent	31	13	18		

*WLC* waiting list condition, *SST* social skills training

About 58 % of the participants had at least one comorbid diagnosis and 23 % had multiple comorbidities. The most common comorbidity was ADHD, which was seen in 40 %. Anxiety disorders, mood disorder, (parent–child) relational problems, adjustment disorder, disruptive behavior disorder, and tic disorder were also reported, but at lower frequencies. The percentage of participants with comorbidity across the two groups was not significantly different.

Most of the children attended regular education (*n* = 41), whereas the others were in special schools (*n* = 11). The mean age of the total sample was 10.1 years (*SD* = 1.27), and did not differ significantly between the WLC and SST (mean ages being 10.0, *SD* = 1.10 versus 10.2, *SD* = 1.43, respectively; *t*(46.8) < 1). The male/female ratio was also comparable for both conditions (WLC: 23 boys and 3 girls; SST: 24 boys and 2 girls).

In 79 % of participants other types of treatment were used either before or in parallel with the group SST. Non-pharmacological treatments ranged from psycho-education sessions for parents to individual child therapy. In addition, 38 % of the sample received some form of psychoactive medication. Medication use was comparable across the groups: most commonly children received methylphenidate (WLC: 31 %; SST: 35 %), while a minority received methylphenidate and Risperidone (WLC: 4 %; SST: 4 %) or Risperidone alone (WLC: 0 %; SST: 4 %). The type of medication and the dosage were kept stable as far as possible over the group SST and this was achieved in 75 % of the cases. The percentage of participants with an adjustment in medication was similar in both groups (i.e., WLC and SST: 25 %).

The majority of participants (88 %) completed the SST (classified as having attended at least 10 of the 12 training sessions). For these children, outcome data (provided by at least one of the informants) were available for 96 % at PRE, 89 % at POST, and 85 % at the FU assessment.

### Intervention

The protocol for the group SST (Deckers et al. 2013) consisted of 12 weekly 1 hour child sessions and three 1 hour parent sessions. Each SST group consisted of four children with ASD, a trained psychologist who guided the group, and a co-therapist. The children received a workbook including the themes and guidelines as well as the homework for each session. Parents also got a workbook providing an overview of the child sessions and instructions to stimulate generalization.

For each child personal learning goals were formulated prior to the group SST. These learning goals were related to the skills that were trained during the group SST as specified in the manual. Examples included asking a question to an unfamiliar person, joining a group of children for play, and waiting for one’s turn. During the group SST two basic themes were repeatedly and consistently addressed, namely basic social skills (consisting of eye contact, voice volume, distance, and posture), and “one good turn deserves another” (if you are kind to another person, then this person will be more likely to be kind to you in return). In addition, more advanced social skills such as listening, recognizing emotions, asking others, having a conversation, joining a group, responding to rejection, responding to emotions of another person, giving and receiving compliments, saying no, and dealing with bullying were covered in the training.

The sessions were highly structured and made predictable with weekly routines and clear group rules. Each SST group session followed a consistent routine: (1). Welcome and overview (i.e., children received a brief outline of the session); (2). Personal highlights of the past week (i.e., participants sharing experiences); (3). Discussion of children’s homework assignments as conducted during the past week; (4). The new topic for the session was introduced, and concrete step-by-step guidelines are given; (5). The children practiced with each other in role play and were provided with instructions and feedback on how to apply the guidelines thereby focusing on their personal learning goals; and (6). New homework was provided for the upcoming week. A group reward system was used to promote practicing at home, obeying to the group rules, and the achievement of personal goals; and thereby working together to earn and share the reward.

In order to enhance generalization, children received homework and parents were instructed on work to do with their child outside the sessions. Each week the therapist contacted the child and his/her parents by e-mail. In the e-mail the topic of the past session and the accompanying homework were described. In addition, brief feedback was provided about the behavior and skills of the child during the past session and corresponding tips for exercising at home were given. The homework included exercises to practice the new topic and social skills outside the group and to reflect on them. The child and parent reported back on achievements and problems to the therapist prior to the next session. In the parental sessions, the parents were more extensively instructed how they could help their child to apply the new skills.

### Assessment

#### Primary Outcome Measure

The primary outcome measure was a paper-and-pencil version of the social skills observation (SSO) as developed by Barry et al. (2003), which was completed by both parents and teachers. The SSO items refer to specific social skills during greeting (11 items), conversation (14 items), and play (11 items) as well as more general social skills (7 items). Parents and teachers asked to indicate whether or not the child or adolescent engaged in these types of social interactions with other persons. After a positive response, questions about the specific social skills had to be completed, such as ‘Did he/she make eye contact?’, ‘Did he/she ask a social question (about feelings or preferences)?’, ‘Did he/she make a positive statement about the play activity?’, ‘Did he/she remain at an appropriate distance from the other person?’, and ‘Did he/she stay calm if teased?’ For each question parents and teachers had to indicate whether their child did or did not apply the specific skill by either responding with “yes” or “no”, or not applicable. A composite score was calculated by summing the “yes” responses. So far, no study has explicitly examined the psychometric properties of the paper-and-pencil version of the SSO. However, in a recent investigation (Deckers, Muris, & Roelofs, manuscript in preparation), we obtained evidence showing that (a) SSO parent- and teacher SSO scores correlated substantially and in a meaningful way with several measures of social functioning in a sample of ASD, clinical control, and non-clinical children aged 7–11 years, and (b) SSO scores of children with ASD were significantly lower than the scores of children in the non-clinical group, which convincingly supports the concurrent and discriminant validity of this observation-based rating scale. In the current study, Cronbach’s alphas of both the parent (*α* = .92) and the teacher (*α* = .88) version of the SSO appeared to be good.

#### Secondary Outcome Measure

Loneliness was measured by means of a subscale of the Loneliness and Aloneness Scale for Children and Adolescents (LACA; formerly known as the Louvain Loneliness Scale for Children and Adolescents; Marcoen et al. 1987), which was completed by the children. For each of the 12 items (e.g., ‘Making friends is hard for me’ and ‘I feel sad because I have no friends’), children indicated how often the item applied to them, using a Likert scale ranging from never (1) to often (4). A composite score was calculated with higher scores indicating higher levels of loneliness. The internal consistency of the LACA is high (in the present study, Cronbach’s *α* was .90) and the validity is satisfactory (Goossens and Beyers 2002).

#### Moderators

The social anxiety subscale of the Screen for Child Anxiety and Related Emotional Disorders (SCARED-71; Bodden et al. 2009) was used to assess children’s level of social anxiety. The parents had to indicate for 9 items how often their child experienced social anxiety symptoms using a 3-point Likert scale with 0 = almost never, 1 = sometimes, and 2 = often. The reliability and validity of the SCARED-71 are convincing (Steensel et al. 2013b), and this is also true for the social anxiety subscale (see Muris et al. 2000; in the current study, Cronbach’s *α* was .90).

In order to assess the typical ADHD symptoms of inattention, hyperactivity, and impulsivity, the ADHD questionnaire (ADHD-Q; Scholte and van der Ploeg 2005) was administered. Parents had to indicate for 18 items how often their child showed ADHD-related behaviors on a 5-point Likert scale ranging from 0 = never to 4 = very often. The reliability and validity of the AVL are good (Evers et al. 2000). In the present study, Cronbach’s *α* was .90.

The Wish for Social Interaction Scale (WSIS; Deckers et al. 2014) was administered for measuring children’s desire for social interactions with other people. The WSIS consists of 8 closed questions about potential social activities with unknown persons (e.g., “Would you like to have a little chat with this person?” and “Would you like to play with this person?”). For each of these eight questions side-view pictures of faces (boys, girls, men and women) were displayed one by one on the computer screen and the children were asked to answer each of these eight questions for 8 people. A total score was calculated by summing the number of positive responses (range 0–64). The internal consistency of the WSIS in the current study was good (*α* = .92).

The Theory of Mind test-Revised (ToM test-R) of Steerneman and Meesters (2009) was administered in order to assess individual differences in children’s level of ToM. The ToM test-R is a (semi-) structured interview for children containing 36 questions divided in 14 items consisting of stories, questions and tasks. Correct answers are coded as 1 and incorrect answers as 0. The total score of the ToM test-R was calculated by summing the correct answers (range 0–36). The reliability of the total score was moderate, with a Cronbach’s alpha of .67 (see also Steerneman and Meesters 2009).

### Statistical Analyses

Multilevel analyses were used to estimate the change in social skills and the change in loneliness over time in both groups. The social skills as observed by the parents and the teachers and the level of loneliness as indicated by the children were the dependent variables. The results were analyzed according to the intention-to-treat principle. The fact that the participants were nested within their training group and might be more similar was taken into account in the multilevel analyses.

Firstly, between-group analyses were conducted. To examine whether the SST group showed a greater increase of social skills as compared to the WLC group, the change in social skills between the two conditions was compared for the parent and teacher ratings of children’s social skills. More specifically, the change in social skills between PRE and POST in the SST group was compared with the change in social skills between BASELINE and PRE in the WLC group (see Fig. 1). A compound symmetry covariance structure for repeated measures was applied as having the best fit, with time point (coded BASELINE = 0 and PRE = 1 in WLC group, and coded PRE = 0 and POST = 1 in SST group), condition (coded WLC = 0 and SST = 1), and time point x condition as fixed effects. The difference between SST and WLC was represented by the time point x condition interaction in the model. The effect sizes expressed in Cohen’s *r* (Cohen 1988; *r* = √(F/(F + df)) were computed from the multilevel estimates. This between-group analysis was also carried out with loneliness as the dependent variable.

Secondly, the within-subject analyses were conducted. The change in social skills over time within the total sample was analyzed and the hypothesized moderators were tested for parent and teacher ratings of social skills separately. Multilevel analyses with a compound symmetry covariance structure for repeated measures were applied. To test whether initial social anxiety, ADHD symptoms, level of ToM, and the desire for social interaction moderated the change in social skills, the centered SCARED, ADHD-Q, ToM test-R, and WSIS scores (obtained at pre-treatment) and their interactions with time points were added as fixed factors to the model. A backward procedure was applied, in which non-significant predictors were stepwise deleted from the model. A similar within-subject analysis, without moderators, was also carried out with loneliness as the dependent variable.

## Results

### SST versus WLC: Primary Outcome Measure (Hypothesis 1)

**Fig. 2:**
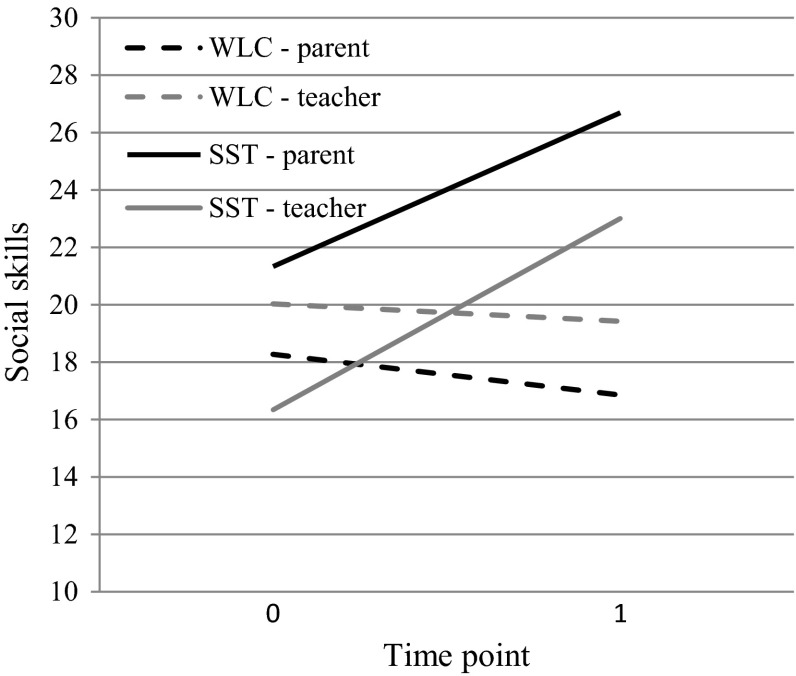
Between-group analyses comparing WLC and SST with regard to parent- and teacher-rated social skills. Note. *WLC* waiting list condition, *SST* social skills training

Table 2 and Fig. 2 show the estimated means on the different time points for both conditions with regard to both the parent- and teacher-rated social skills (primary outcome measure). Table 3 summarizes the results of the accompanying multilevel analysis. With respect to the parent-rated social skills, the Time point × Condition interaction was found to be statistically significant (*p* < .05, *r* = .34), reflecting a greater increase in social skills over time in the SST group as compared to the WLC. The interaction of Time point and Condition was also significant for the teacher-rated social skills (*p* < .01, *r* = .46), again indicating a greater increase of social skills over time in the group SST condition as compared to the WLC. Note that the effect sizes for both interaction effects were in the medium to large range.

**Table 2:** Between-group analyses: Mixed regression-based estimated means comparing parent-rated social skills, teacher-rated social skills, and loneliness between the WLC and SST conditions

Condition	Time point	Social skills parent		Social skills teacher		Loneliness	
		M	SE	M	SE	M	SE
WLC	Time point 0	18.28	2.22	20.03	1.63	24.69	1.56
	Time point 1	16.86	2.26	19.43	1.74	22.72	1.56
SST	Time point 0	21.34	2.19	16.34	1.81	21.42	1.57
	Time point 1	26.69	2.36	23.01	1.93	18.58	1.61

*WLC* waiting list condition, *SST* social skills training

**Table 3:** Results of multilevel analyses comparing the WLC and SST conditions

Mixed model analyses					
	*B*	95 % CI (*B*) (*n* = 26)	T	Df	*P*
*Social skills parent*					
Intercept	26.68	21.98; 31.39	11.33	67.90	<.001
Condition	−9.83	−16.35; −3.31	−3.01	66.43	.004
Time	−5.34	−9.97; −.72	−2.35	34.13	.025
Time point × condition	6.76	.31; 13.22	2.13	33.80	.040
*Social skills teacher*					
Intercept	23.00	19.14; 26.87	11.89	65.31	<.001
Condition	−3.58	−8.77; 1.62	−1.38	64.20	.174
Time point	−6.67	−10.61; −2.72	−3.44	34.54	.002
Time point × condition	7.27	2.19; 12.34	2.92	31.68	.006
*Loneliness*					
Intercept	18.58	15.36; 21.79	11.54	65.23	<.001
Condition	4.15	−.34;8.63	1.85	62.46	.069
Time point	2.84	.71; 4.97	2.69	47.08	.010
Time point × condition	−.87	−3.75; 2.00	−.61	46.29	.544

*WLC* waiting list condition, *SST* social skills training

### SST versus WLC: Secondary Outcome Measure (Hypothesis 2)

In contrast to our expectations, the Time point × Condition interaction for the secondary outcome measure of loneliness was non-significant (*p* = .54, *r* = .09), which indicates that children in the SST condition did not show a larger decrease in loneliness over time as compared to children in the WLC.

### Short-Term and Long-Term Effects of Group SST for the Total Sample (hypotheses 3 and 4)

**Fig. 3:**
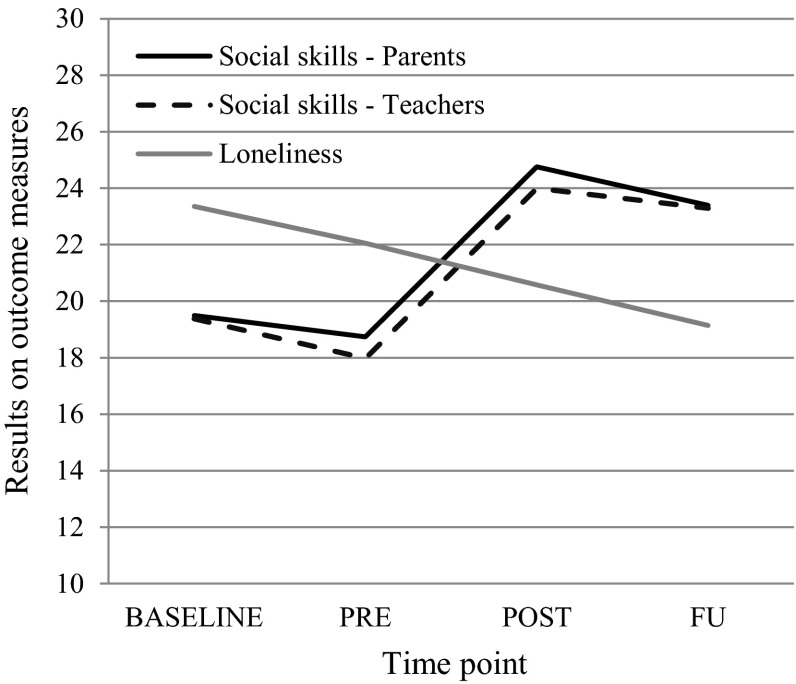
Change in parent- and teacher-rated social skills and child-rated loneliness over time

The multilevel analyses performed on the BASELINE, PRE, POST and FU parent-rated social skills data of the total sample revealed no change between BASELINE and PRE but did indicate a significant increase in social skills between PRE and POST and between PRE and FU (see Table 4; Fig. 3). In other words, according to parents, the group SST produced a significant improvement of children’s social skills, and this positive change was still visible at a follow-up of 3 months. Note that the course in social skills as rated by the teachers showed a highly comparable pattern. That is, no change was observed between BASELINE and PRE, but between PRE and POST children’s social skills clearly increased and this improvement was still present at the FU assessment.

**Table 4:** Results of multilevel analyses comparing the change in social skills and loneliness over time (for parent-rated social skills the centered SCARED and ToM-test R and for teacher-rated social skills the centered SCARED were added as covariates to the model)

Pairwise comparisons (based on estimated marginal means)					
	Mean difference	95 % CI difference	T	Df	*P*
*Social skills parent*					
BASELINE—PRE	.75	−2.69; 4.20	.43	80.42	.665
PRE—POST	−6.02	−8.95; −3.08	−4.08	78.61	<.001
PRE—FU	−4.66	−7.63; −1.69	−3.12	79.25	.003
POST—FU	1.36	−1.76; 4.48	.87	76.68	.387
*Social skills teacher*					
BASELINE—PRE	1.41	−1.79; 4.60	.88	60.66	.382
PRE—POST	−6.01	−9.15; −2.87	−3.83	59.68	<.001
PRE—FU	−5.32	−8.90; −1.73	−2.97	59.91	.004
POST—FU	.70	−3.20; 4.59	.36	61.56	.722
*Loneliness*					
BASELINE—PRE	1.31	−.77; 3.39	1.25	106.59	.215
PRE—POST	1.47	−.25; 3.19	1.70	105.16	.093
PRE—FU	2.91	1.04; 4.78	3.08	106.26	.003
POST—FU	1.44	−.46; 3.33	1.51	104.28	.135

*WLC* waiting list condition, *SST* social skills training

As can be seen in Table 4, the level of loneliness remained fairly stable from BASELINE to PRE. However, between PRE and POST a marginally significant decrease of loneliness was noted, whereas from PRE to FU a significant decline of loneliness was observed.

### Effects of Moderators (Hypothesis 5)

None of the hypothesized variables (social anxiety, ADHD symptoms, ToM, and the desire for social interaction) moderated the change in parent-rated social skills between PRE and POST or between PRE and FU. However, social anxiety [*β* = −4.20, *t*(45) = −3.71, *p* = .001] and level of ToM [*β* = 3.15, *t*(46) = 2.81, *p* = .007] did have a main effect on the level of social skills. Note that the relation between social anxiety and social skills was negative and that between ToM and social skills positive. This indicates that in general higher levels of social anxiety were associated with lower levels of social skills, whereas higher levels of TOM were generally linked to higher levels of social skills.

When using teacher-rated social skills as the outcome variable in the moderator analysis, it was again found that social anxiety, ADHD symptoms, ToM, and the desire for social interaction did not have an effect on the change in social skills over time. Only a main effect of social anxiety was found [*β* = −2.65, *t*(44) = −2.72, *p* = .009] the negative relation again showed that in general higher levels of social anxiety were associated with lower levels of social skills.

## Discussion

The present study evaluated a group SST for high-functioning children with ASD in an outpatient community mental health center. The findings clearly support the effectiveness of this type intervention for children with these pervasive developmental problems. That is, social skills improved significantly according to both parents and teachers, suggesting a successful generalization of the newly learned skills. The follow-up assessment showed that the positive effects were still present at 3 months follow-up. Results revealed that children’s feelings of loneliness did not change to the same degree as their improvement in social skills. This is understandable as the improvement in social skills will not immediately result in higher levels of positive social interactions. Over time, however, it can be expected that the improved skills will lead to a higher frequency of positive encounters with other children, with a consequent reduction in feelings of loneliness. The results of this study also demonstrated that the level of social anxiety, ADHD symptoms, ToM, and desire for social interaction did not moderate the treatment outcome. Altogether, group SST seems suitable for a quite heterogeneous group of (high-functioning) children with ASD.

The present study contributes to the existing research about group SST for children with ASD (Dawson and Burner 2011; Rao et al. 2008; Reichow and Volkmar 2010; Reichow et al. 2012). The study was ecologically sound being based in a regular community mental health center using a subject group which was fairly typical of the referred population. Effort was made to keep balance between a faithful reflection of regular clinical practice and a methodologically sound research design. Clinicians working in daily practice provided the training to representative ASD children and their parents in this mental health care setting. The results of the between-group analyses demonstrated that the increase in social skills can be ascribed to the SST rather than to time or assessment effects. One could argue that parents closely followed the clinical process of their children and hence were not blind to the treatment condition. However, the teachers were not actively involved in the treatment process and thus observed the children with more distance. Even though parents and teachers observed the children in a different context, the pattern of findings was highly similar, which emphasizes the robustness of the results. Although we employed a thorough and detailed diagnostic procedure to classify children with ASD, an obvious limitation of the current study was the absence of standardized diagnostic instruments for autism spectrum problems, such as the Autism Diagnostic Interview-Revised (Lord et al. 1994) or the Autism Diagnostic Observation Schedule (Lord et al. 1999). Note further that diagnoses were made in terms of the DSM-IV-TR (American Psychiatric Association 2000) and that almost two-thirds of the children were classified as having PDD-NOS, a diagnosis that no longer exists in the latest edition of the DSM (DSM-5; American Psychiatric Association 2013). Thus, it is possible that in terms of the current diagnostic criteria, many children of this study would qualify for ‘mild’ to ‘moderate’ ASD or that they may fulfill the criteria for an alternative classification such as social communication disorder (see Smith et al. 2015; Van Steensel et al. 2015). Please note that the core deficits in ASD are social communication and interaction and this still plays a major role in the DSM-5 categories for which social skills training is of paramount importance. In addition, as the group SST was administered as a component of a personalized treatment plan, the pure effects of the group SST remain unknown. On the other hand, adjacent treatment(s) were also provided for WLC participants, yet the SST-WLC comparison yielded clear evidence for the effectiveness of SST, at least as reported by teachers and parents.

The experiences and results of the present study may have some implications for clinical practice. In the first place, the results are promising and encourage the implementation of group SST for high-functioning children with ASD. However, in clinical practice different types and variations of group SST are available. There are some specific factors that might have contributed to the success of the present group SST, which may be valuable to consider for other clinicians. In the current group SST, effort was made to optimally match the training context to the specific needs of children with ASD. That is, the sessions had a predictable routine, visual support was used, concrete group rules and concrete step-by-step instructions were provided and consequently applied (Krasny et al. 2003). Within the structured context of the group sessions, there was still some room to attend to individual learning goals and to provide each child with instructions and feedback tailored to his/her own level. Like in other treatment settings, we think that common factors and the therapeutic relationship were important (Lambert and Barley 2001). We invested in a working relationship with the child and its parents and established a secure atmosphere within the group. In addition, two basic themes were repeatedly and consistently addressed (basic social skills and “one good turn deserves another”) and the children were encouraged to implement these principles in multiple situations. It turned out that the children quickly got familiar with these principles and were able to apply this new knowledge in other situations. The involvement of the parents seemed to contribute to this effect as well, as they were explicitly instructed to provide their child with feedback regarding these principles in daily life. We experienced that the weekly e-mail contact intensified the involvement of the parents and the children in several ways: this was an additional contact in-between the group sessions, which helped to stimulate the child to transfer the newly learned skills to their natural environment; parents got an active role and shared responsibility; personalized feedback and instructions were provided so parents could adapt their feedback and support more easily to the level of their child; and the child itself also received additional tips on how to apply the newly learned skills in daily practice.

Future research is needed to further examine the long-term effectiveness of group SST and to explore working mechanisms of this type of intervention, which are responsible for the improvement and generalization of social skills in children with ASD. Although social interaction deficits are a core feature of ASD and are considered to be pervasive, social skills turn out to be accessible and responsive to intervention. We have to realize that even small improvements in social skills may have significant implications in the daily life of a child with ASD and other social communication and interaction problems and his/her surroundings.
